# A Review of Recent Advances in Neuroprotective Potential of 3-N-Butylphthalide and Its Derivatives

**DOI:** 10.1155/2016/5012341

**Published:** 2016-12-08

**Authors:** Idriss Ali Abdoulaye, Yi Jing Guo

**Affiliations:** Department of Neurology, The Southeast University Affiliated Zhong Da Hospital, No. 87 Dingjiaqiao, Nanjing, Jiangsu Province 210009, China

## Abstract

The research of alternative treatment for ischemic stroke and degenerative diseases has always been a priority in neurology. 3-N-Butylphthalide (NBP), a family of compounds initially isolated from the seeds of* Apium graveolens* Linn., has shown significant neuroprotective effects. Previous extensive studies have demonstrated that NBP promotes a better poststroke outcome and exerts a multitargeted action on several mechanisms, from oxidative stress to mitochondrial dysfunction to apoptosis to inflammation. Additionally, recent findings on several neurological disorders have shown that NBP's beneficial effects extend beyond the management of stroke. However, despite the increasing number of studies toward a better understanding and the rapid advances made in therapeutic options, to date, dl-3-N-butylphthalide, a synthetic variation of l-3-N-butylphthalide, remains the only clinically approved anti-ischemic agent in China, stressing the difficulties for a viable and effective transition from experimental to clinical practice. Events indicate that NBP, due to its multitargeted effect and the adaptability of its basic structure, can be an important game changer and a precursor to a whole new therapeutic approach to several neurological conditions. The present review discusses recent advances pertaining to the neuroprotective mechanisms of NBP-derived compounds and the possibility of their clinical implementation in the management of various neurological conditions.

## 1. Introduction

The search for new treatments of neurodegenerative diseases and ischemic stroke has always been the focus in neurology. This is mostly due to their incurability, the complexity of their pathological process, the tremendous medical cost, the psychological burden generated, and finally the resulting postmanagement disability. As more work is being done aiming to better understand their mechanisms, several pathophysiological similarities have been observed on a subcellular level. Those similarities range from increased levels of oxidative stress to mitochondrial dysfunction to excitotoxicity to inflammatory changes to protein aggregation and apoptosis [1–3]. Neuroprotection, as a treatment, aims to, through a multitargeted approach, achieve an effective prevention of disease progression and a minimization of related disabilities, by means of stopping or at least slowing neuronal loss [1].

**Figure 1:**
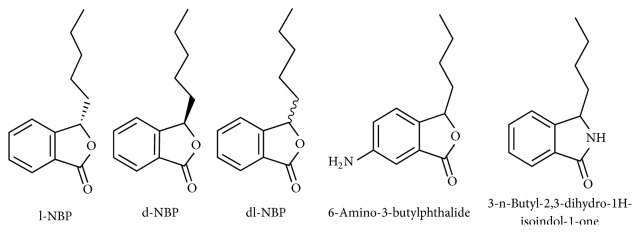
Various structures of NBP and its derivatives.

3-N-Butylphthalide (NBP), a family comprised of optical isomers l-3-N-butylphthalide (l-NBP) and d-3-N-butylphthalide (d-NBP) (Figure 1), with l-NBP being an extract from seeds of* Apium graveolens *Linn. (celery) and dl-3-N-butylphthalide (dl-NBP), a synthetized version, has been studied for its significant neuroprotective effects. Extensive experimental and clinical studies have furthermore confirmed those properties leading to the approval and marketing of dl-NBP as an anti-ischemic drug in China since 2002. Several studies focused on the human metabolism and pharmacokinetics of NBP showed that NBP is safe for human usage [4] and that it essentially undergoes oxidation by cytochrome P450 (P450) after oral administration, resulting in 23 identified metabolites, among which 10-keto-NBP (M2), 3-hydroxy-NBP (M3-1), 10-hydroxy-NBP (M3-2), and NBP-11-oic acid (M5-2) are considered as major metabolites [5, 6]. Those studies also established that hydroxylation of the n-butyl side chain and C-3 constitutes the main metabolic route and that urine is the main excretion pathway of dl-NBP metabolites [6]. Additionally, it has been found that the likelihood of drug-to-drug interaction is minimized by the diversity of enzymes, such as P450, alcohol dehydrogenase (ADH), and aldehyde dehydrogenase (ALDH), involved in its metabolism [6]. However, the participation of 3-hydroxy-NBP sulfate, one of the sulfated metabolites of dl-NBP in the process, is believed to be the main reason for dl-NBP's side effect (mild hepatotoxicity) [7]. Many researches involving stroke animal models have also concluded that NBP, through multiple mechanisms, significantly reduces the cerebral infarct size and oxidative damage, inhibits platelet aggregation, improves blood flow and mitochondrial functions, possesses antithrombotic and anti-inflammatory effects, and reduces neural apoptosis [8–10], hence the importance of understanding its mechanisms and the value it might hold for future management of ischemic stroke as well as neurodegenerative diseases. This article aims to summarize and discuss recent experimental findings related to the mechanisms underlying NBP and its derivatives in ischemic stroke and neurodegenerative diseases. NBP will refer specifically to l-3-N-butylphthalide, d-3-N-butylphthalide, and dl-3-N-butylphthalide in our further writings.

## 2. Methods

The search for articles was performed on PubMed and ScienceDirect databases using keywords such as 3-N-butylphthalide, l-3-N-butylphthalide, dl-3-N-butylphthalide, and NBP. This yielded a wide range of articles including human trials, animal models, and cell cultures. Recent journal articles written in English on animal models or cell cultures directly assessing several or one of the mechanisms and effects of NBP on these conditions: ischemic stroke, neurodegenerative diseases, demyelinating diseases, diabetes, myocardial infarction, and hypertensive nephropathy were selected.

## 3. NBP and Ischemic Stroke

Ischemic stroke is a major health problem and the third leading cause of mortality and physical disability. This implies that stroke prevention and most importantly the identification and protection of ischemic but not yet infarcted brain tissues, the limitation of tissue damage, and the prevention of stroke recurrence virtually define the goal of modern management. It has been demonstrated by previous studies that, apart from reduction of oxidative damage, inhibition of platelet aggregation, and improvement of mitochondrial functions [8–10], NBP also improves stroke outcome by increasing the level of circulating endothelial progenitor cells [11]. Additionally, an in vitro study evaluated the effects of multiple fluoro-, chloro-, and bromoanalogs of NBP by means of resting tension on isolated rat thoracic aorta ring assay, and the result showed that two promising products,* 3-butyl-6-fluoro-1(3H)-isobenzofuranone* and* 3-butyl-6-bromo-1(3H)-isobenzofuranone*, possess enhanced vasorelaxant effects compared to traditional NBP [12]. This shows that new NBP-related designs can be viable and may be prone to further experimental evaluation since both compounds might prove to be good candidates for treatment of the acute phase of ischemic or hemorrhagic stroke. Although NBP's mechanisms of action on ischemic stroke have already been extensively studied, several remain elusive, with new ones still being discovered.

### 3.1. Antiplatelet Aggregation and Antithrombotic Effects

Several experimental studies have shown that NBP possesses an inhibitory effect on platelet aggregation. L-NBP has shown promising antiplatelet properties through an increase of cyclic adenosine monophosphate (cAMP) levels in platelets and an inhibition of serotonin release [13]. However, due to its low potency, several substitutes have been designed and studied for their antiplatelet and antithrombotic effect. The majority displayed promising, sometimes superior effects through several yet unknown mechanisms. Studies on* 3-n-butyl-2,3-dihydro-1H-isoindol-1-one* (Figure 1), a synthetic compound resulting from a coupling of NBP and edaravone, demonstrated a direct inhibitory effect on blood clots formation, and adenosine diphosphate (ADP) and arachidonic acid (AA) induced platelet aggregation [14, 15]. In two other studies, the first involved* 2,5-Bis*{*2-[(S)-(+)-1-(2-(4-methylpiperazin-1-yl)acetoxy)]-pentyl*}*benzoate-1,4 : 3,6-dianhydro-D-glucitol*, an active ring-opened NBP derivative, and the second involved* (±)-(E)-2-methoxy-4-(3-(2-(nitrooxy)ethoxy)-3-oxoprop-1-en-1-yl)phenyl2-(1-(2-(4-(5-(4-(3-thioxo-3H-1,2-dithiol-5-yl)phenoxy)pentyl)piperazin-1-yl)acetoxy)pentyl)benzoate*, an NO/H_2_S-donating derivative, and both compounds exhibited additional benefits such as in vitro nitric oxide (NO) and hydrogen sulfate (H_2_S) production, protection against acute thrombosis, and inhibition of the ischemia/reperfusion-related injury. Furthermore, they produced better neurobehavioral improvement coupled with higher levels of antioxidant SOD, GSH, GSH-Px, and optimal blood-brain barrier (BBB) penetration compared to NBP [16, 17]. This could be crucial for future improvement of poststroke cerebral circulation [16, 18]. Other H_2_S releasing compounds, such as* 6-amino-3-n-butylphthalide *(Figure 1) and* (±)-6-(4-(3-thioxo-3H-1,2-dithiol-5-yl)phenoxy)hexyl 2-(1-acetoxypentyl) benzoate,* also showed superior antiplatelet aggregation and antithrombotic properties compared to NBP, aspirin, or ticlopidine hydrochloride [19], with an additional protection against collagen and adrenaline induced thrombosis [18, 19].

### 3.2. Effects on Mitochondrial Function

The protective effect of NBP on mitochondrial damage after ischemic stroke has been extensively studied. Early animal studies have shown that dl-NBP improves the activities of Na^+^/K^+^-ATPase and Ca2^+^-ATPase in mitochondria. Those activities not only are crucial to the maintenance of cell membrane potential but also participate in the effect transport and regulate cell volume. An increase in the levels of enzyme cytochrome c oxidase was also observed after treatment with dl-NBP on rats model [20]. As it has already been proven, this enzyme is very important for the prevention of chemical asphyxiation of cells and is greatly inhibited during the ischemic stroke process. Another study demonstrated that dl-NBP reduces the release of cytochrome c in the cytosol of mice cell after acute ischemia [21]. This shows that dl-NBP plays a very important role in attenuating caspase-dependent apoptosis. Furthermore, caspase-dependent apoptosis was also evaluated through assessment of mitochondrial ischemic related apoptosis inducing factor (AIF) release. The result was that cytosolic AIF was considerably reduced while mitochondrial AIF was higher [22]. This further supports the idea that dl-NBP attenuates caspase-dependent apoptosis. Another in vitro study assessing the effects of* (S)-ZJM-289*, a novel NO-releasing derivative of NBP, has shown that* (S)-ZJM-289* reduces ROS accumulation and intracellular calcium overload, resulting in an attenuation of OGD/R-induced neuronal injury, a process essential to mitochondrial normal function and integrity [23]. All of the above results suggest that dl-NBP and certain NBP derivative possess a cumulative beneficial effect on the process of mitochondrial damage and exert a direct influence on the release of apoptotic factors related to mitochondrial activity.

### 3.3. Effects on Neuronal Cell Apoptosis

Caspase-3 and caspase-9 are, respectively, members of the cysteine-aspartic acid protease and aspartic acid specific protease family, with both, once activated, playing a central role in the execution-phase of cell apoptosis. Another group, the B cell lymphoma 2 (Bcl-2) family, is comprised of regulator proteins possessing both inhibitory and mediatory functions and is considered as an important antiapoptotic protein. Many previous and recent studies showed that treatment with dl-NBP greatly influences the level of those proteins. An experimental study showed an increase in levels of Bcl-2 and hypoxia-inducible factor 1 alpha (HIF-1*α*) coupled with a decrease of caspase-3 expression [22] after administration of dl-NBP [24]. Another study suggested a new antiapoptotic approach of dl-NBP through activation of phosphatidylinositide 3-kinase (PI3K)/protein kinase B (Akt) signaling pathway [25] and possible regulation of c-Jun N-terminal kinase (JNK) and p38 mitogen-actives protein kinases (MAPK) activation [22]. These studies demonstrated that dl-NBP could be a good candidate for prevention of further cellular death in the ischemic penumbra. Additional protective properties such as amelioration of ischemia reperfusion-induced brain injury have also been attributed to NBP. It is believed that NBP upregulates HGF levels consequently inhibiting the TLR4/NF- kB signaling pathway [26].

### 3.4. Effects on Oxidative Stress

The ischemic cascade is characterized by several mechanisms, among which is oxidative stress. The excess calcium entry, a result of hypoxia and ischemia, causes generation of free radicals, reactive oxygen species, and calcium-dependent enzymes (calpain, endonucleases, ATPases, and phospholipases); this process is referred to as excitotoxicity. Treatment with long-term administration of NBP on stroke model indicated a significant decrease of malondialdehyde (MDA) levels in brain tissues of rats, suggesting that NBP counteracts excitotoxicity and restores the balance by enhancement of antioxidant response [27]. Another study on bone marrow of rat confirmed those findings and further suggested that dl-NBP reduces hydrogen peroxide- (H_2_O_2_-) induced accumulation of reactive oxygen species (ROS) [25]. These findings combined demonstrate that NBP's multiple antioxidant effects can be decisive in the treatment outcome of ischemic stroke patients and might constitute an important field for further therapeutic options.

### 3.5. Effects on Neurogenesis

Since neurogenesis is a critical step toward recovery after ischemia, many studies have been conducted with the aim of understanding its impact on poststroke brain functions recovery. One particular study came to the conclusion that there is a significant correlation between upregulation of neurogenesis and ischemia in some parts of rodent brain [28]. It has been hypothesized that there is a possibility of migration of newborn precursor cells to the damage areas prior to maturation. However, the downfall is that newly generated cells hardly survive [29]. A recent study stated that dl-NBP stimulates the angiogenic process and protects the brain microvascular endothelial cells (BMECs) against oxygen glucose deprivation (OGD), by means of an increase of HIF-1*α* and Bcl-2 expressions [24]. Additionally, studies on newborn neural cells showed that l-NBP improves their proliferation, survival, and differentiation [29]. The underlying process has been described as through an increase of 5-bromo-2′-deoxyuridine- (BRdU-) positive cells in the dentate gyrus of damaged areas. Furthermore, an increase in growth-associated protein-43 (GAP-43) expression, cAMP response element-binding protein (CREB) activity, and synaptophysin was also observed, indicating the possibility of a positive effect of l-NBP on neurogenesis, newborn neurons survival, and neuroplasticity [29]. Recent studies have been more focused on the effects of NBP on tandem-pore-domain potassium channels. Potassium channel subfamily K member 2 (TREK-1), a member of the two-pore-domain potassium, has been shown to exert, through modulation of resting membrane potential, a control effect on neuronal excitability [30]. Previous studies have shown the close correlation between the pathological process of ischemic stroke and the levels of TREK-1 mRNA and protein expressions [31, 32]. Although the mechanism by which TREK-1 expression affects cellular functions remains unclear, it is believed, under hypoxic conditions, that the upregulation of TREK-1 expression inhibits the activity of protein kinase A and the expression of cyclin D1 [33] and decreases neuronal stem cell [34], astrocyte [35], and human osteoblasts proliferation [35, 36]. Recently, it has been demonstrated that l-NBP promotes neurogenesis and tissue recovery through inhibition of TREK-1 channels [37]. Although the specific mechanism by which NBP affects TREK-1 and the resulting long-term effects need to be further studied, TREK-1 might be a new field to explore for future neuroprotection purposes and a good target for new specifically designed NBP derivatives.

In summary, the multitargeted approach of NBP and the fact that new mechanisms are being discovered not only show us that NBP is still not fully understood but also lets us imagine its potential. In addition, its versatility and the broad possibilities of viable engineered for optimal effect compounds make it unique and valuable for the treatment of stroke and possibly neurodegenerative diseases. However, the above-enumerated studies clearly stress the need for a continuous experimentation and a more targeted approach for future NBP-related compounds.

## 4. NBP and Neurodegenerative Diseases

### 4.1. NBP and Alzheimer's Disease (AD)

AD, as the most common form of dementia, is a major, incurable public health problem with no real means of stopping or reversing its progression [38]. The main pathological change in AD has been identified as a protein misfolding caused by plaque accumulation of abnormally folded amyloid beta (A*β*) protein and tau protein in the brain [39].

Several experimental studies have shown that NBP possesses a multitargeted beneficial approach on the pathology of AD. Initial studies showed that both l-NBP and dl-NBP exhibit protective effects against mitochondrial damage through a significant inhibition of A*β*-induced mitochondrial dysfunction and A*β*-induced active caspase-3, caspase-9, and cytochrome c expressions. Additional reduction of mitochondrial membrane potential and ROS production were also observed. This shows that NBP directly influences the process of neuronal cell death and the regulation of antiapoptosis protein Bcl-2 [40, 41]. However, l-NBP additionally inhibited the activation of MAPK and JNK/stress-activated protein kinase signaling pathway [40], a mechanism that has not been yet seen with dl-NBP. Another study on cultured astrocytes showed that dl-NBP exerts partial anti-inflammatory effects by acting as a potential inhibitor of nuclear factor kappa-light-chain-enhancer of activated B cells (NF-*κ*B). It inhibits A*β*-induced activation of astrocytes and suppresses the upregulation of cyclooxygenase-2 (COX-2), prostaglandin E2 (PGE2), tumor necrosis factor alpha (TNF-*α*), and interleukin 6 (IL-6) [42]. Most importantly, dl-NBP inhibits A*β*-induced nuclear factor of kappa light polypeptide gene enhancer in B cells inhibitor, alpha (I*κ*B*α*) degradation, and NF-*κ*B nuclear translocation 1-42 [42]. This finding suggests that dl-NBP plays, through inhibition of the NF-*κ*B signaling pathway, an important role against AD-associated neuroinflammation and astrocytes activation. Abnormal phosphorylated tau in AD brains is recognized as among the central pathological events in a complex neurodegenerative cascade. A double transgenic study on l-NBP indicated a significant improvement in spatial learning and memory deficits and also an inhibition of tau hyperphosphorylation by regulation of key kinase activity [43]. The mechanism is believed to be throughout inhibition of CDK-5 and GSK-3 pathways. In addition, l-NBP treatment decreases aggregated TBS-T-soluble and guanidine-soluble A1-total levels. The underlining mechanism might be through the inhibitory effects of cyclin-dependent kinase-5 (CDK-5) and glycogen synthase kinase 3 (GSK-3) signaling pathways [43]. Further antioxidant properties of dl-NBP such as reduction of ROS levels [41], oxidative damage, and reduction of MDA and protein carbonyl (PCO) have also been seen in an APP/PS1 mouse model of AD. Additionally, an increase in activity of antioxidant enzyme and hippocampal glutathione (GSH) levels, a major protective antioxidant, has also been observed after dl-NBP treatment in both Tg mice and WT mice [44]. It resulted in amelioration of neuronal dystrophy of dendrite and spine in the hippocampi of APP/PS1 mice brain and a reduction in the synaptic loss of neurons [44]. This study suggested that dl-NBP achieves protection against oxidative stress by potentially enhancing the cross talk between CREB and nuclear factor erythroid 2-related factor 2 (Nrf2) via CREB-binding protein (CBP) [44]. Moreover, previous studies showed the crucial role of tropomyosin receptor kinase B (TrKB) associated brain-derived neurotrophic factor (BDNF) signaling in protection against memory impairment and regulation of neurogenesis in the hippocampus of AD [45, 46]. It has also been shown that BDNF and its receptor TrKB were downregulated in early stages of AD [47, 48] and that TrKB downstream enzymes including phosphatidylinositide 3-kinase (PI3K) and protein kinase B (Akt) were inactivated [49, 50]. Consequently, the PI3K/AKT pathway is important for the mediation of neuronal survival under many conditions. A study involving APP/PS1 mice demonstrated that l-NBP significantly increases the expression of BDNF/TrkB/PI3K/AKT [49]. Furthermore, l-NBP has shown to be able to restore synaptic and spine function possibly through its effects on the Wnt/b-catenin pathway [51]. These findings show that NBP plays a crucial role in prevention of neuronal loss and memory impairment and, more importantly, in the regulation of neurogenesis in AD models.

### 4.2. NBP, Vascular Dementia (VaD), and Vascular Cognitive Impairment without Dementia

Vascular dementia is a preventable condition that encompasses a group of syndromes with various mechanisms. Several promising studies have been performed to determine whether NBP can be used for the management of dementia. Since the mechanisms by which NBP might be effective in vascular cognitive impairment without dementia are similar to those of VaD, we will focus on VaD in our further writings. Previous research showed that the main mechanisms of VaD are related to the postcleavage release of amyloid precursor protein, A*β* 1-40 and A*β* 1-42, by actions of A*β* enzyme (beta-site APP cleaving enzyme (BACE) or *β*-secretase) and *γ*-secretase [52], and that BACE and A*β* can be indicators for VaD severity [53, 54]. NBP is believed to improve VaD management through its antioxidant, antiapoptotic effects and through reduction of A*β* deposits. Recent researches on animal models either of AD, VaD, or human neuroblastoma cells have shown that l-NBP achieves, through reduction of amyloidogenic A*β*, increase of alpha amyloid precursor protein (*α*-APP) levels (a product of *α*-secretase that is essential in the production of nonamyloidogenic A*β*) and its effect on tau hyperphosphorylation levels and inhibitory effects on neurofibrillary tangles formation, resulting in suppression of neurofibrillogenesis [55]. Other attributes to l-NBP are the reduction of white matter lesions and apoptotic cell death in the hippocampus and frontal cortex [55]. The same study showed that since A*β* production and oxidative stress are interdependent, l-NBP antioxidant effects can possibly decrease malondialdehyde (a product of lipid peroxidation) levels and astrocyte activation and increase superoxide dismutase (SOD) activity, achieving an antioxidative outcome [55]. Other studies on mice concluded that both l-NBP and dl-NBP improve learning memory [56, 57], ameliorate cerebral hypoperfusion, and improve cerebral blood flow [57, 58]. Additionally,* potassium 2-(1-hydroxypentyl)-benzoate*, the predrug of dl-NBP, showed a protective effect against loss of white matter density by reduction of SOD and lipid peroxide activities [59]. Not only did it drastically improve behavioral and morphologic damage in mice but also it increased the expression of phosphorylated Akt-473, a major player in neuroprotection after cerebral ischemia. The mechanism is by affecting the phosphatidylinositol-3 kinase/Akt pathway in the hippocampus [60]. Another study demonstrated that dl-NBP slows the loss of choline acetyltransferase-positive neurons and reduces the mRNA expression of choline acetyltransferase (ChAT), respectively, in the medial septal nucleus, vertical limb, hippocampus, and cerebral cortex of SAMP8 mice [60]. Furthermore, l-NBP has been shown to increase the expressions of *β*-secretase and hyperphosphorylated tau in the hippocampus and parietal lobe of aged rats [61]. These above findings indicate that dl-NBP achieves, through prevention of the central cholinergic system decline, protective effects against age related memory and learning deficit [60]. In summary, all these experimental researches are pointing toward a multitargeted and definitely beneficial effect of NBP in long-term management of dementia prompting for further supportive and more clinical explorations.

### 4.3. NBP and Parkinson's Disease (PD)

PD is a chronic disorder, second to Alzheimer's disease in the family of neurodegenerative diseases. The main pathological changes in PD are degeneration of dopaminergic neurons, Lewy bodies, and accumulation of *α*-synuclein [62, 63]. Few studies assessing the effectiveness of NBP on PD have been performed. A recent experimental study involving PC12 cells indicated that dl-NBP prevents 1-methyl-4-phenylpyridinium-induced (MPP+-induced) neurotoxicity through stimulation of autophagy mediated *α*-synuclein degradation [64, 65]. Another study on rotenone showed that dl-NBP ameliorates SH-SY5Y cell survival against rotenone and improves mitochondrial membrane potential (MMP) reduction and ROS generation and apoptosis. Additionally, dl-NBP can also balance the rotations of apomorphine- (APO-) evoked and SOD activities reduction and even increase vesicular monoamine transporter 2 (VMAT2) levels without influencing alpha-synuclein (SNCA) expression [66]. Furthermore, dl-NBP reduces ROS regeneration and plays an important protective role in mitochondrial functions, integrity, and membrane potential [41]. These above studies are suggesting that dl-NBP can be effective option for the treatment of PD. However, due to the lack of clinical and the scarcity of experimental studies, this remains a promising field to explore.

### 4.4. NBP and Amyotrophic Lateral Sclerosis (ALS)

ALS is a common motor neuron disease with an average survival period of 3 years from onset of weakness [67]. Although very few studies pertaining to NBP and ALS are available, recent tendencies indicated that dl-NBP could, through its multitargeted approach, improve its outcome. Experimental models showed that dl-NBP suppresses the expression of TNF-*α* and nuclear factor *κ*B (NF-*κ*B ) p65 and increases the protein level of nuclear factor erythroid 2-related factor 2 (Nrf2) and heme oxygenase-1 (HO-1) [10]. Since Nrf2 activation reduces postinsult neuronal damage by promotion of anti-inflammatory and prosurvival genes [68], this indicated that dl-NBP could extend survival, improve performance, decrease motor neurons loss, and reduce astrocytosis and microglial activation.

## 5. NBP and Other Conditions 

Preliminary studies have been performed on the effect of NBP on various conditions with systemic cerebrovascular and cardiovascular repercussion such as diabetes, myocardial infarction, or hypertensive nephropathy [69–71]. The results showed several common neuroprotective mechanisms ranging from reduction of oxidative stress to prevention of apoptosis to induction of autophagy through suppression of the *β*-catenin signaling pathway [72] to promotion of angiogenesis. The converging points of NBP antioxidative [71], antiapoptotic, and proangiogenic effects on those conditions are believed to be through reduction of caspase-3 activity, upregulation of vascular endothelial growth factor (VEGF) expression, and elimination of ROS production [70, 73–75]. In addition, it has been indicated that l-NBP possesses additional properties such as the ability of increasing the Bcl-2 protein levels while decreasing cytochrome c levels. More importantly, it inhibits the nucleus translocation of glyceraldehyde-3-phosphate dehydrogenase (GAPDH) protein [69] effectively protecting against ischemia/reperfusion-induced apoptosis. In summary, NBP, due to its multitargeting mechanisms and the increasing discovery of commonalities between several neurological and systemic conditions, might have a very broad use in the future management of various vascular-related diseases.

Demyelinating diseases are common neurological conditions associated with a wide range of underlying etiologies. Recently, it is believed that both mitochondrial dysfunction and apoptosis might play a bigger role in the demyelinating process [76] and that NBP improves its outcome. Possible mechanism is believed to be through improvement of mitochondrial energy pump and activities of mitochondrial complex 4 coupled with a downregulation of JNK pathway [77]. Another possible mechanism might be through protection of medullary sheath by antiapoptotic effects [77]. However, further investigation of those mechanisms is needed.

## 6. Conclusion

Taking into account the seriousness of physical disability and financial burden generated and the death toll created by neurodegenerative diseases and stroke combined, the search for new therapeutic approach is more than necessary. However, the complexity of the pathological process of those conditions makes the development of new therapeutic options a tremendous challenge. The same reason also makes the topic of neuroprotection equally challenging. Several previous attempts involving a wide range of neuroprotective compounds have produced unsatisfactory results whether on experimental or clinical trials and have since been abandoned. NBP, traditionally used for the management of ischemic stroke, has proven to possess a multitargeted approach and to be effective not only in stroke but also in many conditions such as dementia or demyelination. NBP and its derivatives have been essentially shown to possess antiplatelet aggregation, antithrombotic, antiapoptotic, and antioxidant properties coupled with promotion of neurogenesis and protection of mitochondrial functions. Further studies on new designs of NBP derivatives, although lacking clinical follow-ups, have yielded viable, effective, and more targeted compounds. More importantly, these findings indicate how essential NBP's multitargeted approach, the difference in properties between isomers and its structural adaptability, can be for future treatments, since NBP has the potential to be a precursor to a whole new group of drugs and therapeutic approach to several neurological conditions. This opens a new perspective for future treatment of a wide range of conditions. The potentiality is even greater since several possible mechanisms of NBP have yet to be explored. We believe that NBP will play a crucial role in future treatments.

## Abbreviations

NBP: 3-N-Butylphthalide
cAMP: Cyclic adenosine monophosphate
ADP: Adenosine diphosphate
AA: Arachidonic acid
NO: Nitric oxide
BBB: Blood-brain barrier
H_2_S: Hydrogen sulfide
AIF: Apoptosis inducing factor
Bcl-2: B cell lymphoma 2 family
HIF-1*α*: Hypoxia-inducible factor 1 alpha
PI3K: Phosphatidylinositide 3-kinase
Akt: Protein kinase B
JNK: c-Jun N-terminal kinase
MAPK: p38 mitogen-actives protein kinases
MDA: Malondialdehyde
H_2_O_2_: Hydrogen peroxide
ROS: Reactive oxygen species
OGD: Oxygen glucose deprivation
BMECs: Brain microvascular endothelial cells
BRdU: 5-Bromo-2′-deoxyuridine
GAP-43: Growth-associated protein-43
CREB: cAMP response element-binding protein
AD: Alzheimer's disease
A*β*: Amyloid beta
NF-*κ*B: Nuclear factor kappa-light-chain-enhancer of activated B cells
COX-2: Cyclooxygenase-2
PGE2: Prostaglandin E2
TNF-*α*: Tumor necrosis factor alpha
IL-6: Interleukin 6
I*κ*B*α*: Nuclear factor of kappa light polypeptide gene enhancer in B cells inhibitor, alpha
CDK-5: Cyclin-dependent kinase
GSK-3: Glycogen synthase kinase 3
PCO: Protein carbonyl
GSH: Hippocampal glutathione
Nrf2: Nuclear factor erythroid 2-related factor 2
CBP: CREB-binding protein
BDNF: Brain-derived neurotrophic factor
TrKB: Tropomyosin receptor kinase B
PI3K: Phosphatidylinositide 3-kinase
VaD: Vascular dementia
BACE: Beta-site APP cleaving enzyme
*α*-APP: Alpha amyloid precursor protein
SOD: Superoxide dismutase
ChAT: Choline acetyltransferase
PD: Parkinson's disease
MPP+: 1-Methyl-4-phenylpyridinium
MMP: Mitochondrial membrane potential
APO: Apomorphine
VMAT2: Vesicular monoamine transporter 2
SNCA: Alpha-synuclein
ALS: Amyotrophic lateral sclerosis
HO-1: Heme oxygenase-1
VEGF: Vascular endothelial growth factor
GAPDH: Glyceraldehyde 3-phosphate dehydrogenase
P450: Cytochrome P450
ADH: Alcohol dehydrogenase
ALDH: Aldehyde dehydrogenase.
